# Bioinspired nanovesicles released from injectable hydrogels facilitate diabetic wound healing by regulating macrophage polarization and endothelial cell dysfunction

**DOI:** 10.1186/s12951-023-02119-3

**Published:** 2023-10-03

**Authors:** Weiyue Zhang, Xueyang Yang, Xin Huang, Lulu Chen

**Affiliations:** 1 Department of Endocrinology, Union Hospital, Tongji Medical College, Huazhong University of Science and Technology, Wuhan, 430022 China; 2 Hubei provincial Clinical Research Center for Diabetes and Metabolic Disorders, Wuhan, 430022 China; 3 Department of Orthopaedics, Union Hospital, Tongji Medical College, Huazhong University of Science and Technology, Wuhan, 430022 China

**Keywords:** Bioinspired nanovesicles, 4-octyl itaconate, Macrophage polarization, Neovascularization, Diabetic wound healing

## Abstract

Wound healing is one of the major global health concerns in diabetic patients. Overactivation of proinflammatory M1 macrophages could lead to delayed wound healing in diabetes. 4-octyl itaconate (4OI), a derivative of the metabolite itaconate, has aroused growing interest recently on account of its excellent anti-inflammatory properties. Cell membrane coating is widely regarded as a novel biomimetic strategy to deliver drugs and inherit properties derived from source cells for biomedical applications. Herein, we fused induced pluripotent stem cell-derived endothelial cell (iEC) membrane together with M1 type macrophage membrane to construct a hybrid membrane (iEC-M) camouflaged 4OI nanovesicles (4OI@iEC-M). Furthermore, bioinspired nanovesicles 4OI@iEC-M are incorporated into the injectable, multifunctional gelatin methacryloyl hydrogels for diabetic wound repair and regeneration. In our study, bioinspired nanovesicles could achieve dual-targeted deliver of 4OI into both M1 macrophages and endothelial cells, thereby promoting macrophage polarization and protecting endothelial cells. With the synergistically anti-inflammatory and immunoregulative effects, the bioinspired nanovesicles-loaded hydrogels could facilitate neovascularization and exhibit superior diabetic wound repair and regeneration. Taken together, this study might provide a novel strategy to facilitate diabetic wound healing, thereby reducing limb amputation and mortality of diabetes.

**Supplementary Information:**

The online version contains supplementary material available at 10.1186/s12951-023-02119-3.

## Introduction

Diabetes, a chronic metabolic disease, has affected millions of people in the world. Nowadays, about 25% of diabetic patients are afflicted with a remarkable risk of chronic nonhealing wounds including diabetic foot ulcers (DFUs), thereby leading to the outcomes of amputation [1]. Unlike normal wounds, diabetic wounds are often hard to heal due to disrupted glucose and lipid metabolism, ischemia and hypoxia, and persistent inflammatory response [2, 3]. Diabetic wounds are largely different from normal wounds, especially from the aspect of wound microenvironment. In diabetic wound microenvironment, bacterial growth is apt to occur due to the moist skin lesion, high glucose and disordered immunity [4]. Inflammation plays a physiological protective role of the body, which could defend against further injury and remove damaged tissue. Whereas, uncontrolled inflammation hinders wound healing in diabetic patients, including dysregulated macrophages, neutrophils, and so on [5]. In diabetic wound microenvironment, the pro-inflammatory phenotype (M1) macrophages continue to dominate with insufficient amount of the anti-inflammatory phenotype (M2) macrophages [6]. Macrophages take part in every stage of wound healing and repair by avoiding infection, removing damaged tissue, and secreting cytokines. Prolonged M1 activation or imbalanced M1/M2 activation is observed with delayed tissue repair [7]. Then, the persistently dominant M1 macrophage-induced prolonged inflammatory response exerts significant damage to endothelial function and angiogenesis and delays wound healing [8, 9].

Macrophage remains the most attractive therapeutic target for reducing inflammation and promoting chronic wound healing. The treatment strategies that might induce M2 macrophage polarization and prevent inflammation have attracted growing interest. It has been recently reported that metabolic intermediates played a significant role in tissue repair [10]. 4-octyl itaconate (4OI), a derivative of itaconate, has attracted growing interest recently owing to its excellent anti-inflammatory properties [11]. 4OI might alkylate the protein kelch-like ECH-associated protein 1 (KEAP1). Then, the anti-inflammatory transcription factor Nrf2 disassociates from KEAP1 [12], which functions as an important regulator of the level of antioxidants [13]. Meanwhile, a 4OI-loading dynamic Ag–S coordinative multifunctional hydrogel (Ag-SH-PEG) [14] and 4OI-modified black phosphorus nanosheets [15] have been recently studied in diabetic wound repair and have manifested the ability to enhance wound healing. Therefore, 4OI is a promising therapeutic substance for diabetic wound repair. However, the previous trial of 4OI delivery is very limited and lacks targeting ability. Thus, a well-established delivery system is urgently warranted to deliver 4OI into M1 macrophages effectively and sustain its antioxidant and anti-inflammatory effects.

Cell membrane coating as a biomimetic strategy could grant biomaterials with characteristics and functions from source cells [16–18]. Cell membrane-camouflaged nanovesicles, owing to their membrane antigens and structure, possess functions like homologous targeting, long blood circulation, and immune escaping [19, 20]. Hybrid membranes from different cell types are camouflaged on the surface of biomaterials to obtain various functionalities [21, 22]. Jiang et al. fused the red blood cell (RBC) membrane with the cancer cell membrane and constructed the erythrocyte-cancer hybrid membrane-coated nanovesicles [22]. These nanovesicles successfully retained the parent membrane proteins and obtained the functions of longer circulation and homologous tumor targeting simultaneously. To achieve dual-regulation of macrophage polarization and endothelial cell dysfunction, macrophage-targeting and endothelium-targeting are both required. Induced pluripotent stem cell (iPSC)-derived endothelial cells (iECs) exhibited a high degree of similarity with endothelial cells (ECs) in EC-associated genes expression [23]. Bioinspired nanovesicles from iECs reserved the membrane characteristics of iECs, which possessed the feature of ECs [24] and were thus promising in homologous targeting. Therefore, iEC membrane and M1-type macrophage membrane are utilized in our study.

In this study, we fused iEC membrane with M1-type macrophage membrane to construct a hybrid membrane (iEC-M) camouflaged 4OI nanovesicles (4OI@iEC-M). Accordingly, bioinspired nanovesicles could dual-targeted deliver 4OI into both M1 macrophages and endothelial cells, thereby promoting macrophage polarization and protecting endothelial cells. Furthermore, bioinspired nanovesicles 4OI@iEC-M are incorporated into the injectable, multifunctional gelatin methacryloyl (GelMA) hydrogels for diabetic wound repair and regeneration. This study might provide a novel strategy to facilitate diabetic wound repair and consequently reduce limb amputation and mortality of diabetes.

## Results and discussions

### Preparation and characterization of itaconate-loaded bioinspired nanovesicles 4OI@iEC-M

**Fig. 1:**
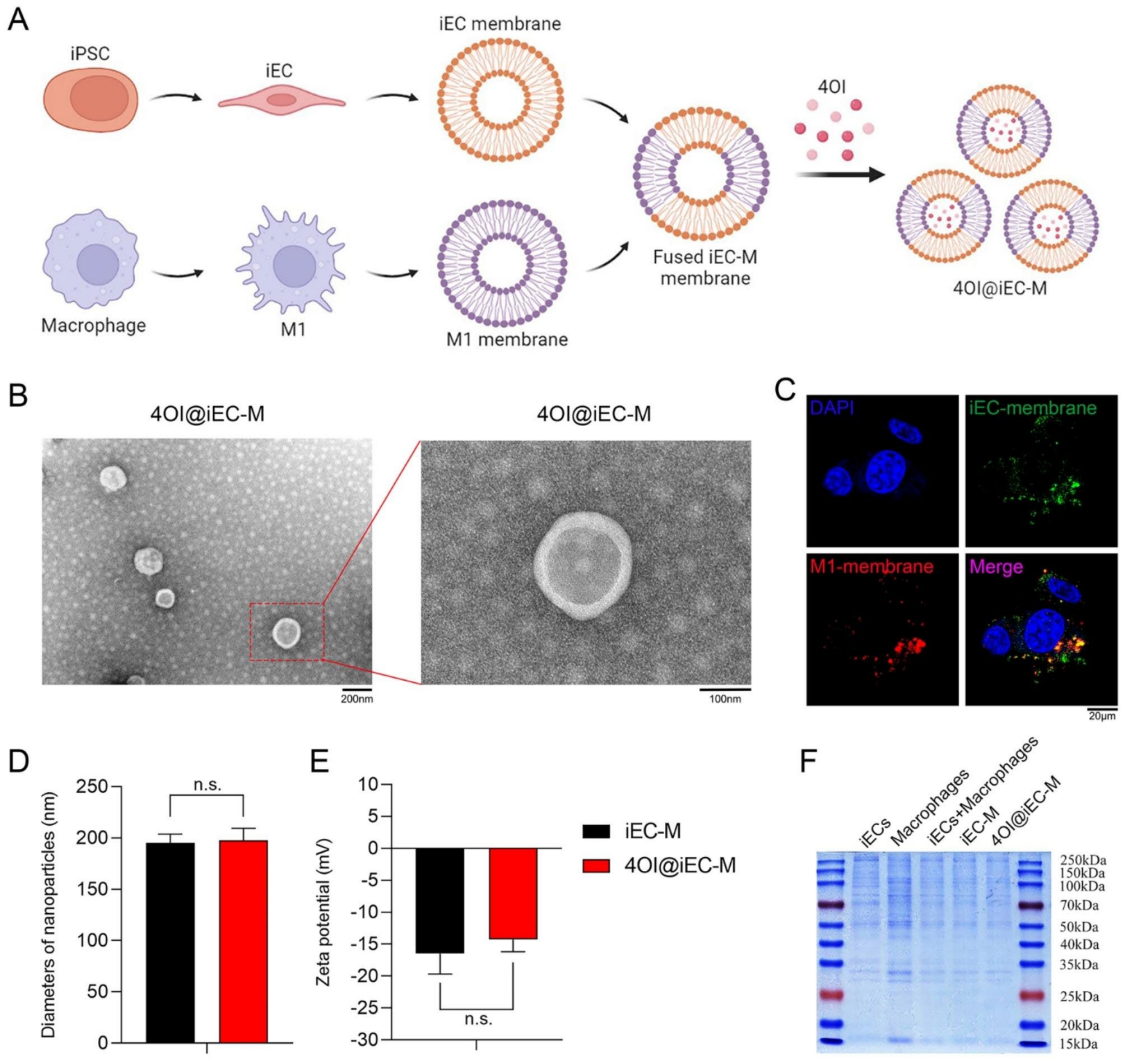
Preparation and characterization of itaconate-loaded bioinspired nanovesicles 4OI@iEC-M. **(A)** Schematic illustrations of the 4OI@iEC-M preparation. **(B)** TEM image of 4OI@iEC-M (scale bar: 200 nm and 100 nm). **(C)** The colocalization of fluorescence in cells derived from FITC (iEC membrane) and DiD (M1 membrane) dyes of iEC-M group (scale bar: 20 μm). **(D)** The characterization of DLS demonstrated that iEC-M and 4OI@iEC-M had similar diameters of around 200 nm (n.s.: no significance). **(E)** The surface zeta potential of iEC-M and 4OI@iEC-M (n.s.: no significance). **(F)** SDS-PAGE was conducted to compare the protein profile of iECs, M1, iEC + M1 cells, iEC-M, and 4OI@iEC-M.

Cell membrane coating nanotechnology has attracted growing interest in the field of targeted delivery strategies, which simultaneously obtain the advantages and functions of both cell membranes and delivered drugs [17]. Compared with extracellular vesicles or exosomes [25, 26], cell membrane-coated nanovesicles are characterized by high output, low cost, simple operation, and good product homogeneity. Accordingly, cell membrane coating nanotechnology is becoming the most promising delivery system. However, monotypic cell membranes could not satisfy the growing demands of clinical applications due to the dynamic and diverse pathophysiological mechanisms in diabetic wounds. The combination of different types of cell membranes might function as multifunctional biomimetic nanoplatforms [27], and could broaden the range of targeting [28]. In this study, the preparation of iEC-M hybrid membrane-coated 4OI bioinspired nanovesicles 4OI@iEC-M was shown (Fig. 1A). We cultured and collected iECs and M1-type macrophages. Furthermore, iEC-M hybrid membrane-derived nanovesicles were constructed via the established extrusion method [27, 28].

Transmission electron microscopy (TEM) images of 4OI@iEC-M showed a characteristic core-shell structure (Fig. 1B). The size of 4OI@iEC-M was around 200 nm. The iEC membrane was labeled with FITC (green) and the M1 membrane was labeled with DiD (red). After iEC-M nanovesicles were incubated with cells, the remarkable colocalization in cells was shown by the fluorescence derived from FITC and DiD dyes of iEC-M group (Fig. 1C). Accordingly, it confirmed the hybrid fusion of cell membranes from two different cell types. Dynamic light scattering (DLS) results indicated that the size of 4OI@iEC-M was around 197.6 ± 11.9 nm (Fig. 1D). The surface zeta potential (Fig. 1E) of 4OI@iEC-M was − 14.3 ± 1.9 mV, similar to that of iEC-M vesicles (-16.5 ± 3.2 mV), which validates the successful fabrication of cell membrane-camouflaged nanovesicles. The SDS-PAGE results showed that iEC-M and 4OI@iEC-M manifested almost the same membrane protein profile as iEC + M1 cells, and incorporated the membrane protein profiles of both iEC and M1 cells (Fig. 1F), thereby further proving the successful fabrication of hybrid membrane vesicles.

### Dual-targeting ability of 4OI@iEC-M for macrophages and endothelial cells

Macrophages play a crucial role in the detection and phagocytosis of “invaders” [29, 30]. Macrophage membrane-coated nanoparticles are endowed with the targeting ability of inflammatory lesions via the CCR2-CCL2 axis [31, 32]. Therefore, macrophage membrane coating nanotechnology could be applied in targeting inflammatory lesion sites and cytokine neutralization. It is reported that the retinal endotheliocyte membranes are equipped with homologous targeting and binding capability to vascular endothelial growth factor (VEGF) [33]. Another study prepared a biomimetic membrane derived from brain microvascular ECs [34], which obtained the homologous targeting capability of ECs. Cui et al. [35] generated bioinspired nanovesicles from iECs, which could mimic the source cells with abundant C-X-C motif chemokine receptor 4 (CXCR4) on the surfaces. And these characteristics equipped the nanovesicles with endothelial homologous targeting ability.

**Fig. 2:**
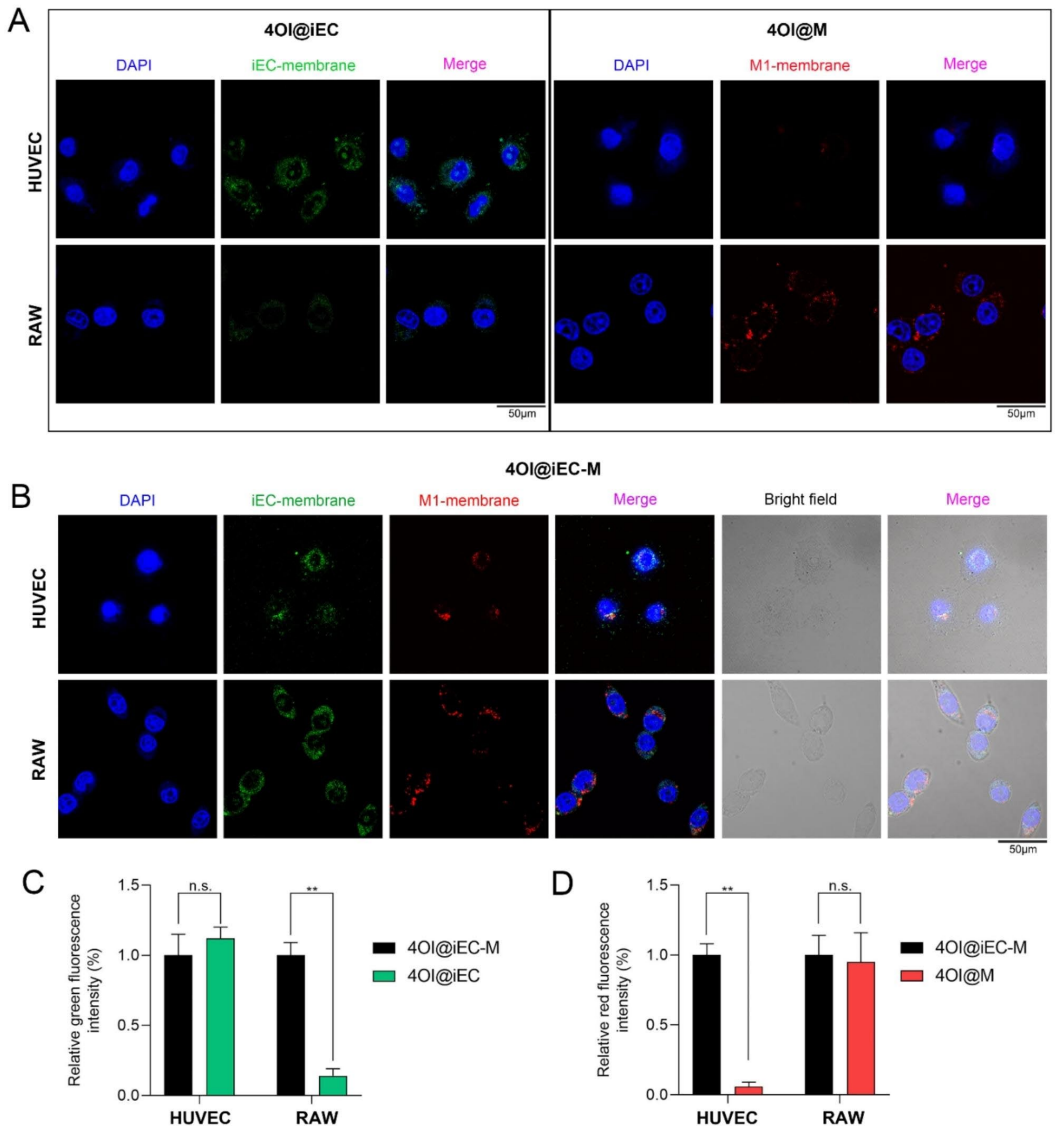
Dual-targeting ability of 4OI@iEC-M for macrophages and endothelial cells. **(A)** The iEC membrane was labeled with FITC (green) and the M1 membrane was labeled with DiD (red). The fluorescence images of HUVEC and RAW cells were shown after incubation with 4OI@iEC or 4OI@M (scale bar: 50 μm). **(B)** The fluorescence images of HUVEC and RAW cells were shown after incubation with 4OI@iEC-M (scale bar: 50 μm). **(C)** The relative green fluorescence intensity of HUVEC and RAW cells after incubation with 4OI@iEC and 4OI@iEC-M (**p < 0.01, n.s.: no significance). **(D)** The relative red fluorescence intensity of HUVEC and RAW cells after incubation with 4OI@M and 4OI@iEC-M (**p < 0.01, n.s.: no significance)

We further explored the dual-targeting effects of 4OI@iEC-M. Hybrid membrane nanovesicles could reserve cell membrane proteins and thus obtain the functions of source cells [28]. We incubated HUVECs and RAW cells with 4OI@iEC, 4OI@M and 4OI@iEC-M respectively, and the fluorescence intensity was examined (Fig. 2A and B). The iEC membrane was labeled with FITC (green) and the M1 membrane was labeled with DiD (red). Both 4OI@iEC and 4OI@iEC-M treatments led to strong green fluorescence intensity in HUVECs, while only 4OI@iEC-M treatment led to strong green fluorescence intensity in RAW cells (Fig. 2C). This suggested that the simple iEC membrane could target HUVEC rather than RAW cells, while hybrid membrane nanovesicles could target both cell types. Similarly, both 4OI@M and 4OI@iEC-M treatments gave rise to strong red fluorescence intensity in RAW cells, while only 4OI@iEC-M treatment led to strong red fluorescence intensity in HUVECs (Fig. 2D). This suggested that the simple macrophage membrane could target RAW cells rather than HUVECs, and hybrid membrane nanovesicles targeted both. Thus, the hybrid membrane nanovesicles exhibited dual-targeting effects.

According to the limited studies utilizing membrane-camouflaged nanovesicles in wound healing, only RBC or platelet membrane was used and they focused on infectious model and anti-bactericidal activity [36, 37]. Besides, RBC and platelet membranes lack targeting ability. Our study is the first that utilized hybrid membrane nanovesicles in diabetic wounds and the proven dual-targeting effects granted the nanovesicles with multiple functionalities.

### Bioinspired nanovesicles loaded injectable hydrogels 4OI@iEC-M/GelMA

**Fig. 3:**
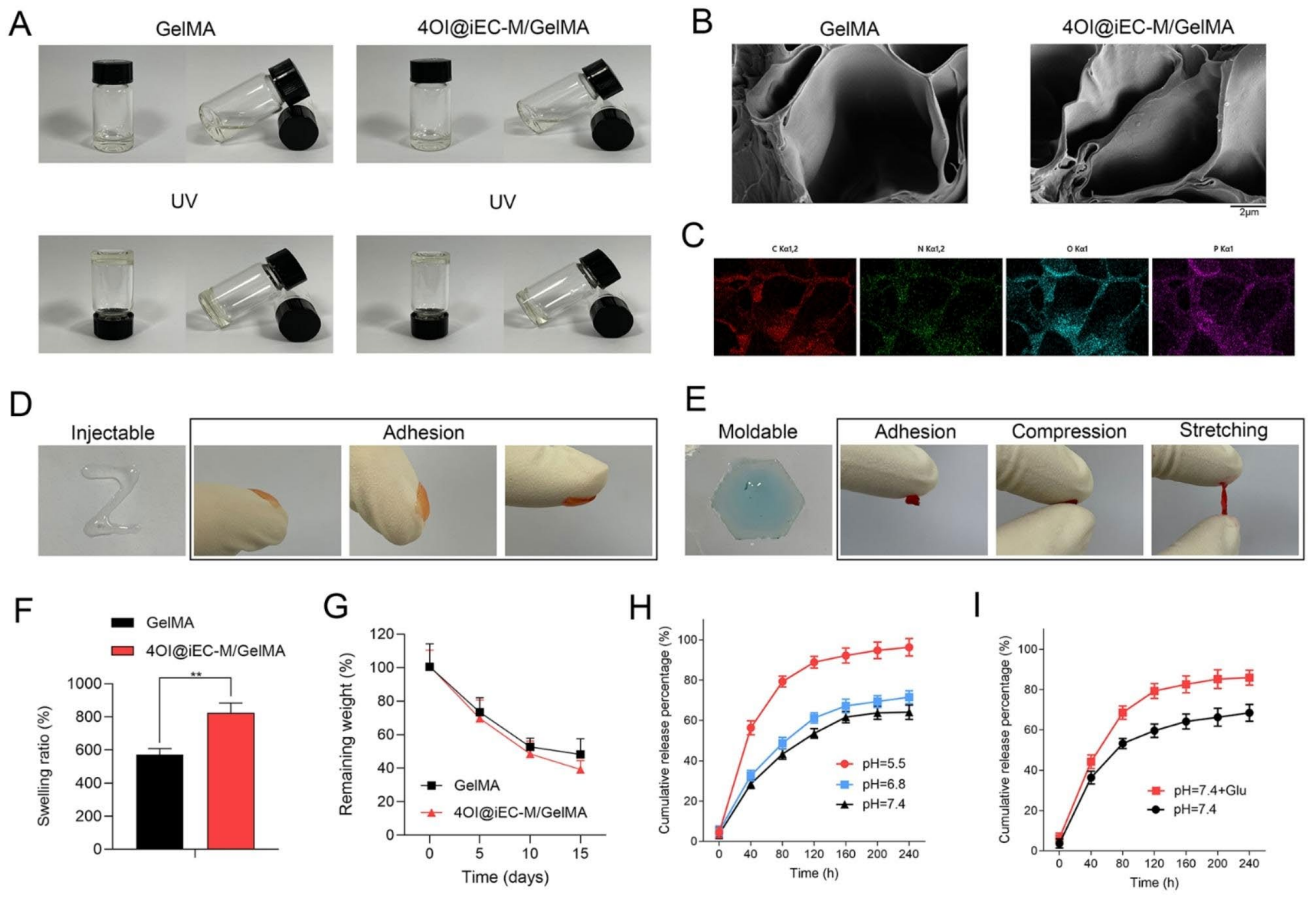
Bioinspired nanovesicles loaded injectable hydrogels 4OI@iEC-M/GelMA. **(A)** The preparation process of injectable hydrogels of GelMA and 4OI@iEC-M/GelMA via under light with a wavelength of 405 nm. **(B)** SEM images of 4OI@iEC-M/GelMA hydrogel (scale bar: 2 μm). **(C)** Elemental mapping images of 4OI@iEC-M/GelMA hydrogel. **(D)** Injectable property and adhesion performance of 4OI@iEC-M/GelMA. **(E)** Remodeling property and representative pictures of 4OI@iEC-M/GelMA hydrogel for adhesion, compression, and stretching. **(F)** The swelling ratios and **(G)** the degradation rates of the GelMA and 4OI@iEC-M/GelMA hydrogels (**p < 0.01). The release behavior of 4OI@iEC-M from the hydrogel in **(H)** an acid environment and **(I)** high concentrations of glucose

GelMA is regarded as a representative hydrogel formulation in biomedical applications. The biomaterials-combined GelMA hydrogels are supposed to be the next-generation platforms for tissue regeneration [38, 39]. During the gelation process (Fig. 3A), the GelMA or 4OI@iEC-M/GelMA solutions were crosslinked under light with a wavelength of 405 nm for 30 Sec [40]. Scanning electron microscopy (SEM) showed the pore morphology of hydrogels (Fig. 3B). The GelMA and 4OI@iEC-M/GelMA hydrogels had a distinct porous structure. No difference in the pore size of the two groups was observed. This pore of hydrogel was important in nutrient exchange during wound healing. As shown in the high-resolution SEM (Fig. 3B), we could observe the nanovesicles in hydrogel, which confirmed that the 4OI@iEC-M was successfully loaded in the hydrogel. 4OI@iEC-M/GelMA elemental mapping images showed that C, N, and O element signals were homogeneously dispersed in the hydrogels, which further indicated that 4OI@iEC-M was loaded in the GelMA hydrogels (Fig. 3C).

The injectability of the 4OI@iEC-M/GelMA was investigated. The hydrogel injected from an injection syringe was utilized to draw “Z”. Appropriate tissue adhesion is critical for wound dressings. It showed that the 4OI@iEC-M/GelMA hydrogel can adhere tightly to fingers without release (Fig. 3D). In Fig. 3E, the spherical hydrogel could be remolded to the hexagonal hydrogel. After compression and stretching, 4OI@iEC-M/GelMA hydrogel could recover to the original state with good flexibility. The swelling ratios of the GelMA and 4OI@iEC-M/GelMA in PBS showed the same trend, with about 825% in the 4OI@iEC-M/GelMA group (Fig. 3F). Therefore, the introduction of 4OI@iEC-M nanovesicles elevated the swelling ratios, which promote wound healing by absorbing body fluids. All the hydrogels degraded with time and the remaining weight was around 40–50% after 15 days (Fig. 3G).

The drug loading efficiency and the drug loading capacity of 4OI@iEC-M were investigated [41] and shown in Supplementary Fig. 2. Reacting 0.024 mg 4OI with 0.1 mg iEC-M (4OI/iEC-M = 0.24) was deemed to be the best condition for preparing 4OI@iEC-M, considering both loading efficiency and loading capacity of the drug. The release behavior of 4OI@iEC-M from GelMA was further detected. 4OI@iEC-M had a remarkable release within 80 h and a slow but steady release over 240 h. The release of the 4OI@iEC-M/GelMA group was remarkably increased in the medium of pH = 5.5 rather than pH = 6.8 or pH = 7.4 (Fig. 3H). Moreover, the release of the 4OI@iEC-M/GelMA group was remarkably increased in the high glucose medium (Fig. 3I). Osmotic pressure and pH are important for maintaining cell homeostasis and cell membrane selective permeability. The strong acid or high glucose environment might destroy the components of cell membranes, leading to the loss of selective permeability of cell membranes. Therefore, the release of 4OI@iEC-M could be accelerated in diabetic wounds with an acidic and high glucose environment.

### Regulative effects of 4OI@iEC-M/GelMA on inflammation and macrophage polarization

**Fig. 4:**
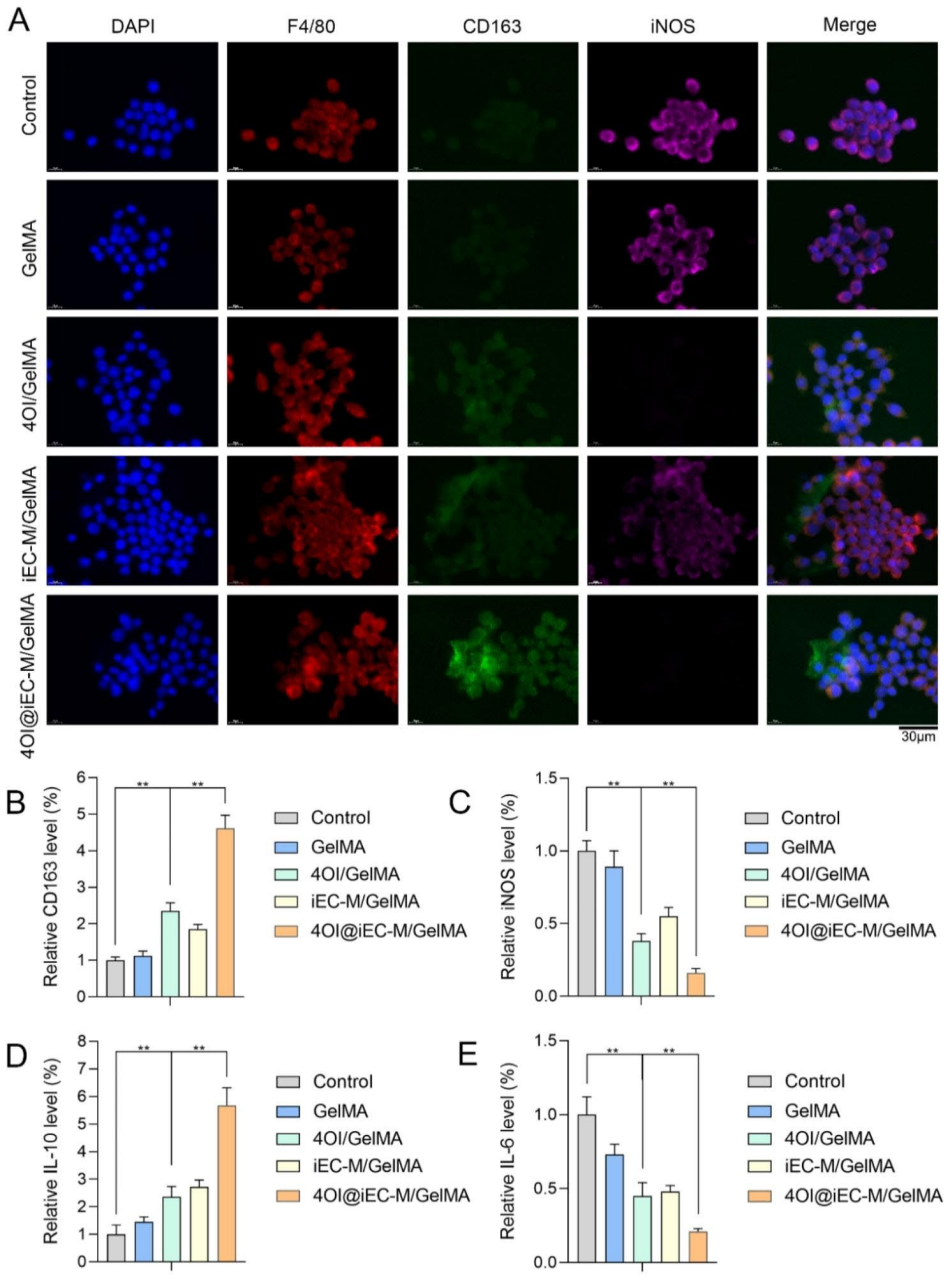
Regulative effects of 4OI@iEC-M/GelMA on inflammation and macrophage polarization. **(A-C)** The proportions of M2 type (F4/80+, CD163+) and M1 type (F4/80+, iNOS+) macrophages in the control, GelMA, 4OI/GelMA, iEC-M/GelMA, and 4OI@iEC-M/GelMA groups (**p < 0.01, scale bar: 30 μm). **(D)** The protein level of M2 cytokines (IL-10) was detected by ELISA (**p < 0.01). **(E)** The protein level of M1 cytokines (IL-6) was detected by ELISA (**p < 0.01)

The prolonged inflammatory phase is the major challenge in diabetic wound healing. We investigated the regulative effects of 4OI@iEC-M/GelMA on inflammation and macrophage polarization under high glucose palmitic acid treatment. The treatment was given with cells on the hydrogel systems. The proportions of M2 type macrophages (F4/80+, CD163+) in the 4OI@iEC-M/GelMA group were remarkably higher than in the 4OI/GelMA group, and the proportion of M1 type macrophages (F4/80+, iNOS+) was downregulated (Fig. 4A-C). The results verified the trend that M2/M1 macrophage ratios in the 4OI@iEC-M/GelMA group were the highest among all groups. The expressions of M2 cytokines (anti-inflammatory IL-10) and M1 cytokines (pro-inflammatory IL-6) were investigated via enzyme-linked immunosorbent assay (ELISA) (Fig. 4D and E). The protein level of IL-6 was reduced, whereas that of IL-10 was increased in the 4OI@iEC-M/GelMA group more than in the other groups. The result suggested that 4OI@iEC-M/GelMA hydrogel might promote wound healing by inflammation regulation.

In normal wounds, M1-phenotype predominates in macrophages from day one to day three of wound healing and then the transition to M2 macrophages begins and peaks around day seven; while in diabetic wounds, the predominating phenotype is constantly M1, which leads to impaired wound repair and weakened angiogenesis [6]. The regulative effects of 4OI@iEC-M/GelMA on the proportions of M1 and M2 macrophages make this system promising not only in inflammation regulation but also in the consequent angiogenesis improvement.

### 4OI@iEC-M/GelMA promotes the functions and angiogenesis of endothelial cells

**Fig. 5:**
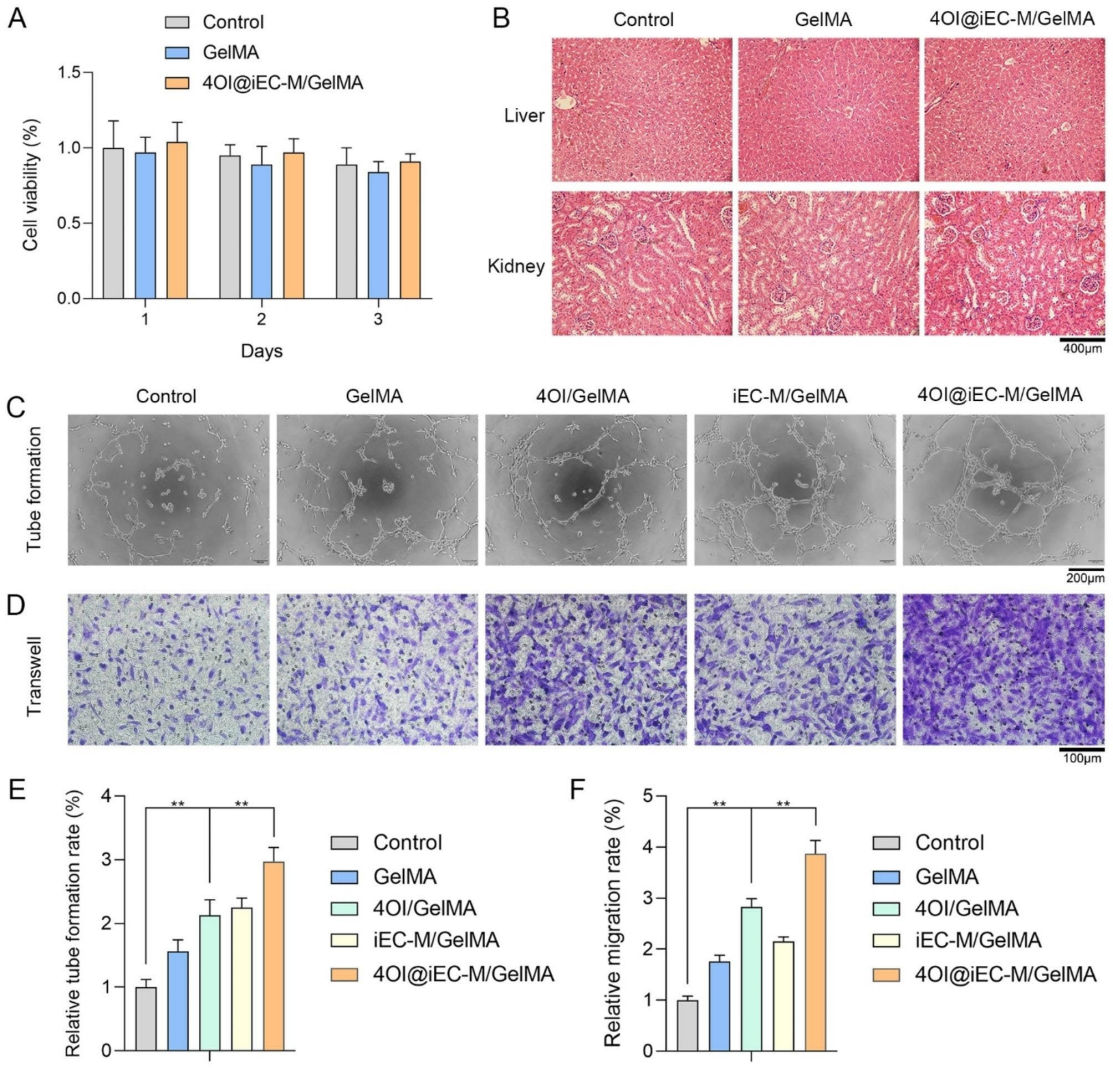
4OI@iEC-M/GelMA promotes the functions and angiogenesis of endothelial cells. **(A)** The viability of HUVECs after incubation with control, GelMA, or 4OI@iEC-M/GelMA for 1, 2, and 3 days. **(B)** The HE staining of liver and kidney of the animal models in this study on wound repair day 9 (scale bar: 400 μm). **(C, E)** 4OI@iEC-M/GelMA group showed increased HUVEC tube formation (6 h) than other groups (**p < 0.01, scale bar: 200 μm). **(D, F)** Transwell assay (24 h) indicated that 4OI@iEC-M/GelMA group showed a higher migration rate than other groups (**p < 0.01, scale bar: 100 μm)

We further evaluated the biocompatibility of this delivery system in vitro and in vivo. Figure 5 A indicated the viability of HUVECs after incubation with control, GelMA, or 4OI@iEC-M/GelMA for 1, 2, and 3 days. Figure 5B indicated the HE staining of liver and kidney of the animal models in this study on wound repair day 9. No obvious cell or organ toxicity was observed in this study. Newly formed vessels are supposed to deliver nutrition and oxygen into wounds, which greatly affects the curative rates of wounds [42]. Based on this, endothelial functions and angiogenesis are critical in wound healing. 4OI@iEC-M/GelMA group showed increased HUVEC tube formation than 4OI/GelMA group, with more tube-like structures and numbers (Fig. 5C and E). Transwell assay revealed that 4OI@iEC-M/GelMA had an elevated migration rate compared with 4OI/GelMA (Fig. 5D F). Taken together, 4OI@iEC-M/GelMA remarkably facilitated the functions and angiogenesis of HUVECs under high glucose and palmitic acid treatment. Enhanced functions and angiogenesis of endothelial cells can promote diabetic wound healing. It has been reported that promoting the angiogenic function of HUVECs could accelerate diabetic wound healing [43]. Thus, the positive in vitro results of 4OI@iEC-M/GelMA in HUVECs indicated that this system might also play a crucial role in enhancing angiogenesis and wound repair in vivo.

### 4OI@iEC-M/GelMA promotes diabetic wound healing in vivo

**Fig. 6:**
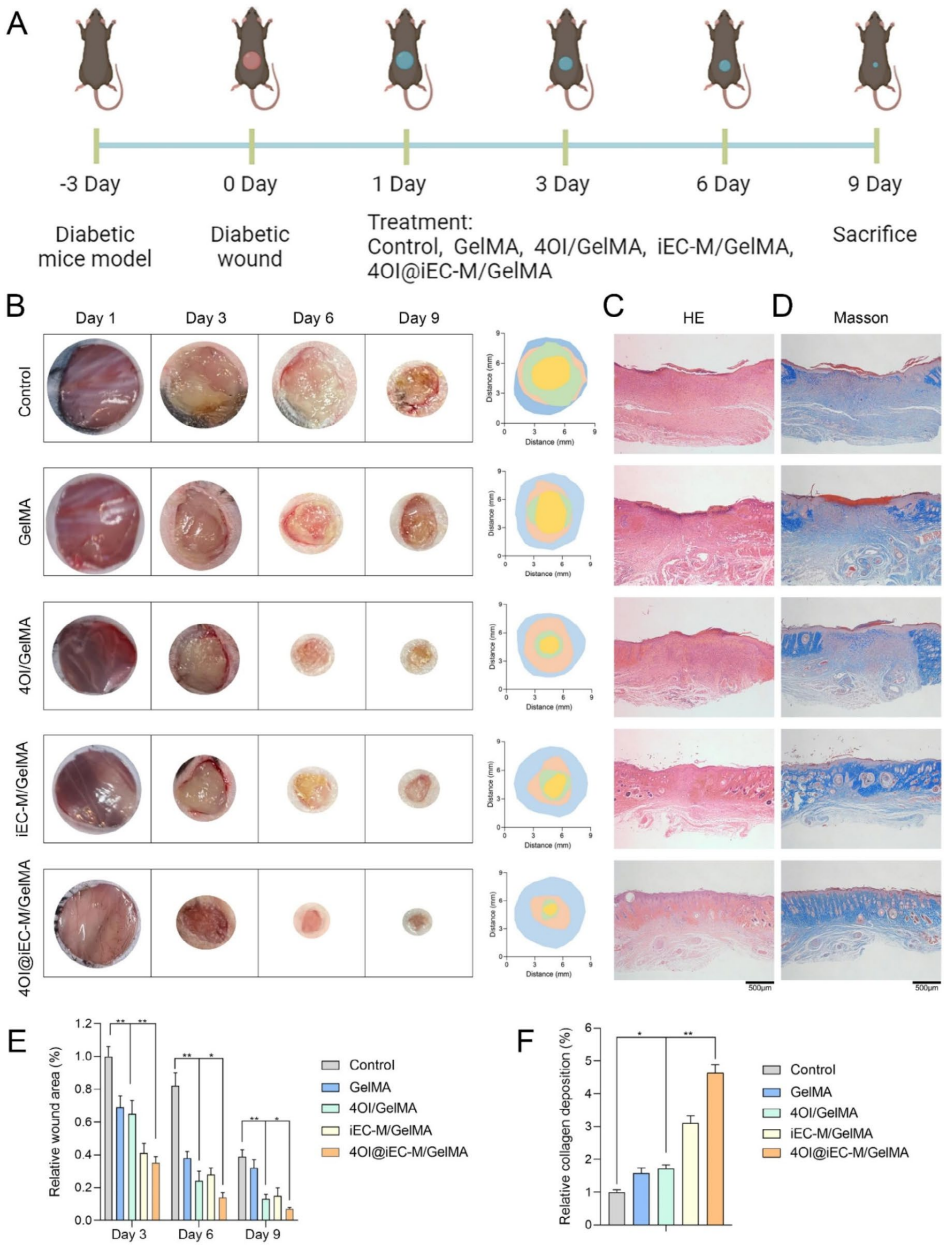
4OI@iEC-M/GelMA promotes diabetic wound healing in vivo. **(A)** The therapeutic strategy of diabetic wound model in vivo. **(B, E)** The macroscopic images of wounds and the corresponding wound area at different time points (**p < 0.01, *p < 0.05). **(C)** The H&E images showed the formation of the epidermis and dermis tissue in diabetic wounds on day 9 (scale bar: 500 μm). **(D, F)** The Masson staining images showed the collagen deposition level of diabetic wounds on day 9 (**p < 0.01, *p < 0.05, scale bar: 500 μm)

We further investigated the regulative effects of 4OI@iEC-M/GelMA in promoting diabetic wound repair in vivo. Figure 6 A depicts the therapeutic strategy. Figure 6B and E showed the macroscopic images of wounds and the corresponding wound area at diverse time points. On day 9, the wound in the 4OI@iEC-M/GelMA group was almost completely healed, while poor wound closure remained in Control and GelMA groups. These indicated that the 4OI@iEC-M/GelMA hydrogel could significantly facilitate diabetic wound healing.

The wound during the healing process was further explored. All the groups were observed with newly formed epidermis on day 9, which was more natural and mature under 4OI@iEC-M/GelMA than other systems (Fig. 6C). H&E staining indicated that 4OI@iEC-M/GelMA treatment led to a better wound healing rate with improved regeneration of epidermis and dermis tissue. As was shown in Fig. 6D F, 4OI@iEC-M/GelMA treatment led to more collagen deposition (blue) than other treatments. Accordingly, the 4OI@iEC-M/GelMA hydrogel improved diabetic wound repair by enhancing the regeneration of epidermis and dermis tissues and increasing collagen deposition.

### Inflammation and angiogenesis regulation effects in vivo

**Fig. 7:**
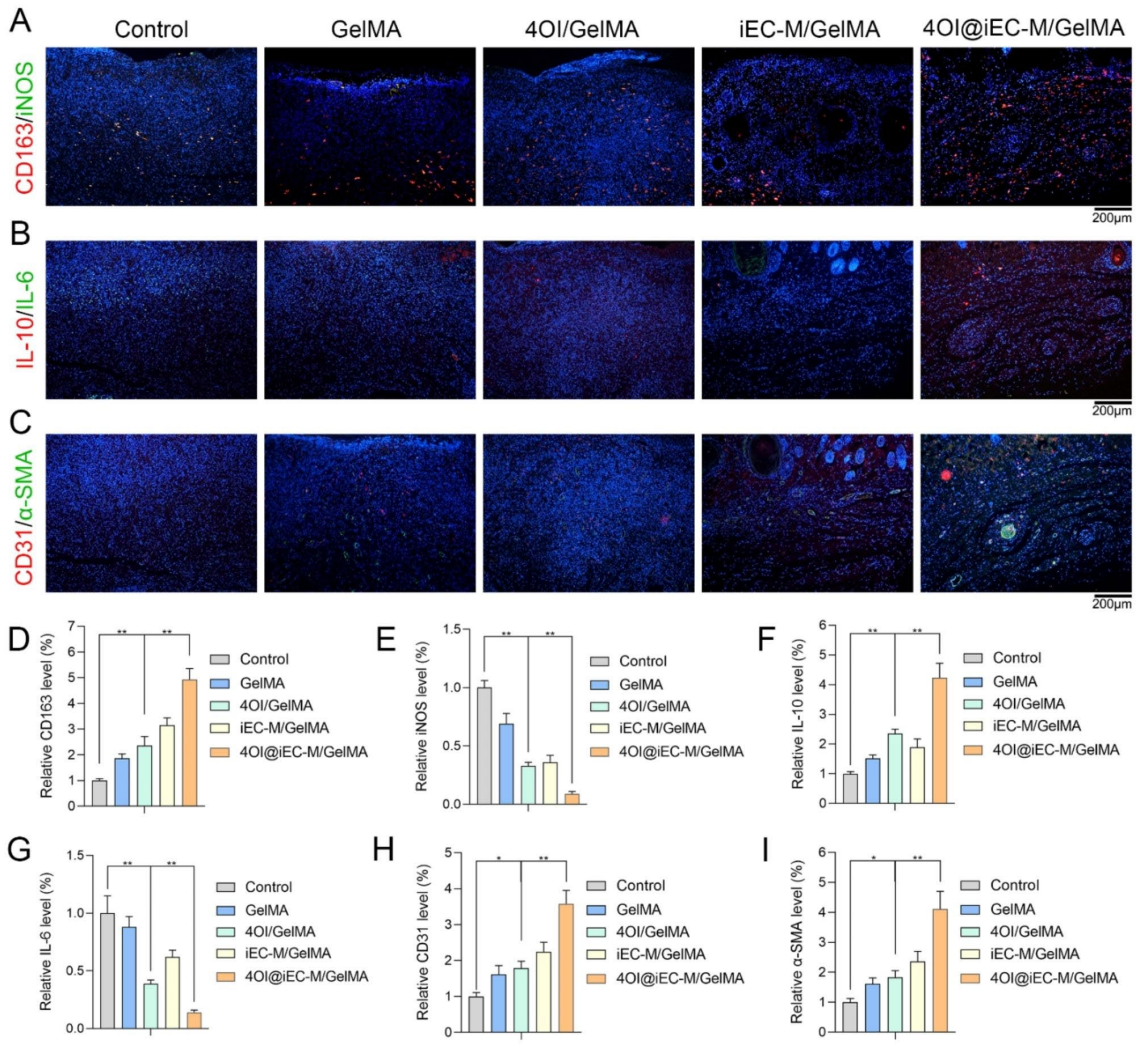
Inflammation and angiogenesis regulation effects in vivo. Tissue sections of the wound area on day 9 were utilized to examine the inflammation and angiogenesis regulation effects in vivo. **(A, D, E)** Double immunofluorescence staining of tissue sections of wound area for CD163 (M2 marker, red) and iNOS (M1 marker, green) (**p < 0.01, scale bar: 200 μm). **(B, F, G)** Double immunofluorescence staining of IL-10 (red) and IL-6 (green) (**p < 0.01, scale bar: 200 μm). **(C, H, I)** Double immunofluorescence staining of CD31 (red) and α-SMA (green) (**p < 0.01, *p < 0.05, scale bar: 200 μm)

Macrophages are of great importance in skin repair during wound healing [44], which includes proinflammatory M1 and anti-inflammatory M2 macrophages. Double immunofluorescence staining investigated the proportions of M2 (CD163) and M1 macrophages (iNOS) (Fig. 7A). More M2 and fewer M1 macrophages were observed in the 4OI@iEC-M/GelMA group (Fig. 7D and E), which showed a reduced inflammatory response in diabetic wounds. The pro-inflammatory factor IL-6 and the anti-inflammatory factor IL-10 are also associated with inflammation in wounds. The least IL-6 and the highest IL-10 expression were shown in the 4OI@iEC-M/GelMA group, indicating few signs of inflammation (Fig. 7F and G). These results demonstrated that 4OI@iEC-M/GelMA hydrogel might effectively decrease inflammation and further promote diabetic wound healing.

Angiogenesis is vital in the remodeling stage of wound healing [45]. M2 macrophages might secrete VEGF and promote the proliferation of ECs in wounds [46]. CD31 is a typical marker of ECs during angiogenesis. Double immunofluorescence staining of CD31 and α-smooth muscle actin (α-SMA) was utilized to analyze neovascularization formation (Fig. 7C). 4OI@iEC-M/GelMA treatment remarkably increased expressions of CD31 and α-SMA than other treatments (Fig. 7H and I). Therefore, 4OI@iEC-M/GelMA hydrogel had improved pro-vascularization ability, thereby effectively promoting diabetic wound healing.

## Conclusions

**Fig. 8:**
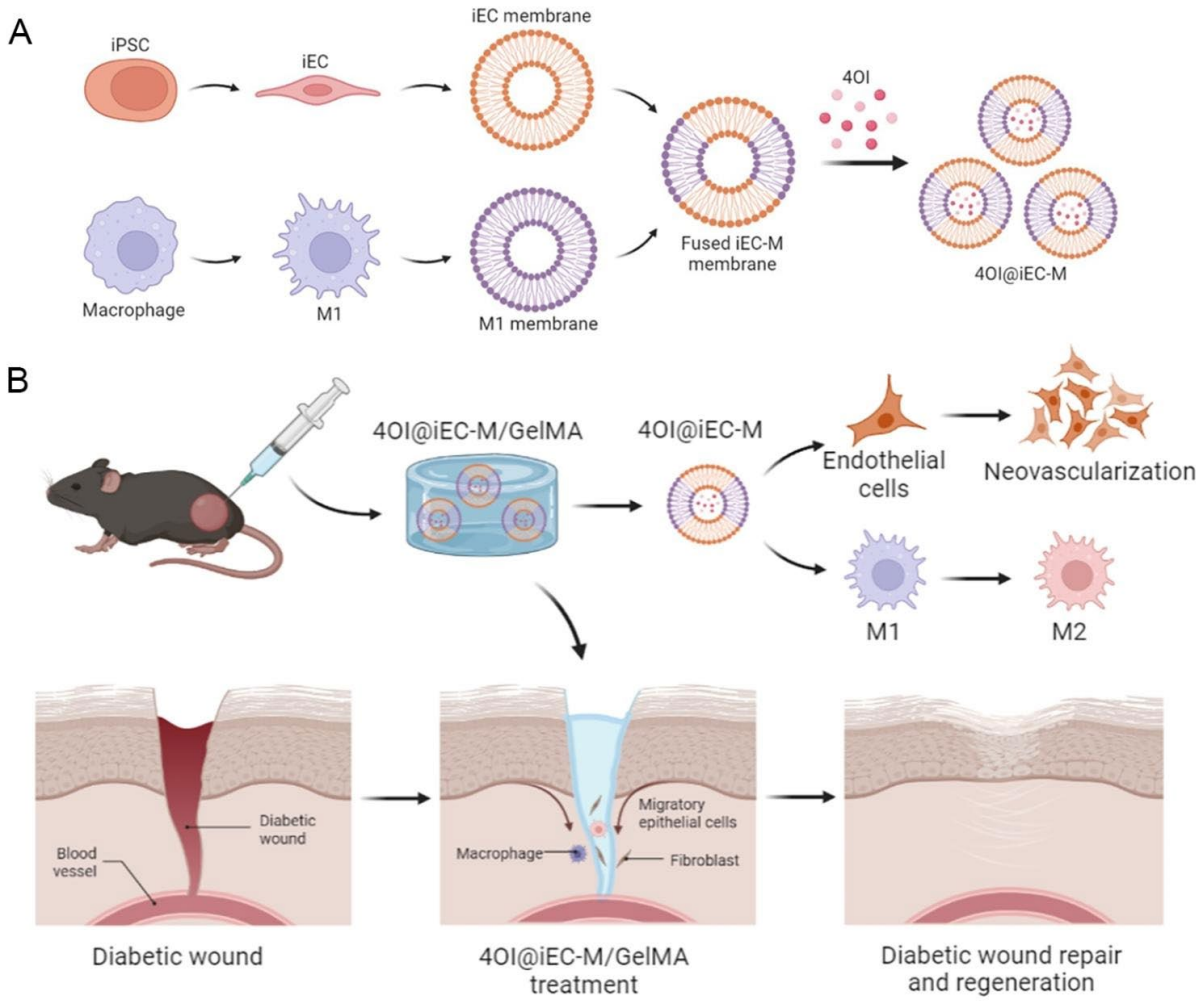
Overview of bioinspired nanovesicles released from injectable hydrogels facilitating diabetic wound repair and regeneration by inflammation regulation. Created with BioRender.com

In summary, we constructed a hybrid membrane camouflaged 4OI nanovesicles (4OI@iEC-M). Furthermore, bioinspired nanovesicles 4OI@iEC-M are incorporated into a multifunctional injectable hydrogel for diabetic wound repair and regeneration (Fig. 8). Owing to 4OI, the hydrogel could induce macrophage polarization to play an anti-inflammatory role. The hydrogel with injectable and self-healing abilities could significantly facilitate the HUVECs functions and accelerate diabetic wound healing by inducing macrophage polarization and angiogenesis.

## Materials and methods

### Materials

4OI was purchased from MedChemExpress (Shanghai, China). GelMA hydrogel (EFL-GM-60) was acquired from EFL (Suzhou, China). FITC, DiD and DAPI were purchased from Solarbio (Beijing, China). LPS was acquired from Cell Signaling Technology (USA). IL-4 was acquired from PeproTech (USA). Primary antibodies iNOS and CD163 were bought from Proteintech. HUVEC and RAW264.7 cells were acquired from Wuhan Warner Biotechnology Co., Ltd (Wuhan, China). The Endothelial Cell Medium (ECM) was purchased from Sciencell (USA). The ELISA kit was bought from Bioswamp (Wuhan, China). The live/dead assay was bought from Beyotime (China). Matrigel 356,234 was purchased from Corning (USA).

### Synthesis of hybrid membrane-derived vesicles

Cell membranes derived from iEC or M1-type macrophages were collected according to established protocols [47]. Around 5 × 10^7^ cells were resuspended with hypotonic lysis A buffer from the membrane extraction kit at 4 °C. Then the membrane solution was disrupted with a homogenizer and differentially centrifuged to purify the membrane. 50 µl of the mixture of iEC membrane (1 mg/ml) and M1 membrane (1 mg/ml) was diluted to 1 ml and then extruded through 200 nm polycarbonate porous membrane to promote membrane fusion [22]. The hybrid membrane-derived nanovesicles iEC-M was obtained for further experiments.

### Synthesis of 4OI@iEC-M

For 4OI loading, 50 µg/ml iEC-M solutions and 4OI (50 µM) were mixed and stirred overnight, and then centrifuged (12,000 rpm, 5 min) to remove free drugs. The 4OI@iEC-M was obtained for further use.

### Synthesis of 4OI@iEC-M/GelMA solutions

The GelMA hydrogel is injectable, and it can be injected by using a syringe and then crosslinked by using UV light at 405 nm (photoinitiator: Lithium Phenyl (2,4,6-trimethylbenzoyl) phosphinate, LAP). 50 µg/ml of 4OI@iEC-M was added in 6% (w/v) GelMA solutions, which were then placed in the dark at 4 °C. For further experiments, the hydrogel solutions were modified into a hydrogel via 405 nm UV light for 30 Sec [40].

### Material characterizations

The morphology of bioinspired nanovesicles 4OI@iEC-M was investigated by a Hitachi TEM system. The size distribution of nanovesicles was analyzed via DLS by a Zetasizer Nano ZS90. SDS-PAGE was conducted to compare the proteins of cells and nanovesicles. Images of the pore structures of the hydrogels were detected via a Zeiss SEM system. Energy-dispersive spectroscopy mapping was used to show the elemental distributions of C, N, P, and O in hydrogels.

### The drug loading and release study

The drug loading efficiency (%) and the drug loading capacity (mg/mg) were defined as described before [41]. Solutions containing 4OI (0.01–0.08 mg) and 0.1 mg of iEC-M was prepared, and then collected by ultra-centrifugation and washed several times until the supernatant became color free. The amount of unbound 4OI in the solution was determined by measuring the absorbance at 199 nm using a UV spectrophotometer. The release behavior of 4OI@iEC-M from GelMA was also investigated via a UV spectrophotometer. 4OI@iEC-M released was investigated within 240 h in acidic solutions (pH = 5.5, pH = 6.8, or pH = 7.4) and high glucose solutions (with pH = 7.4). The UV maximum absorption of 4OI is 199 nm, which is detected for determining 4OI concentrations.

### Targeting ability and in vitro cellular uptake

The iEC membrane was labeled with FITC (green), the M1 membrane was labeled with DiD (red), and the nucleus was stained with DAPI (blue). The HUVEC and RAW cells were incubated with 10 µg/ml 4OI@iEC, 4OI@M, or 4OI@iEC-M for 3 h and were then observed under a confocal laser scanning microscopy (CLSM, Olympus FV1200, Japan).

### Macrophage culture and polarization

The RAW 264.7 cells were grown in DMEM and were exposed to LPS (100 ng/ml) and IL-4 (40 ng/ml) to induce polarization to M1 and M2 phenotypes [48]. Primary antibodies iNOS for M1 and CD163 for M2, were added to the cells and incubated in dark for 30 min at 4 °C. The conjugated secondary antibodies were added and incubated. After washing with PBS, they were observed by CLSM.

### Sodium dodecyl sulfate-polyacrylamide gel electrophoresis (SDS-PAGE)

SDS-PAGE analysis was performed to identify the membrane protein profiles of iECs, M1, iEC + M1 cells, iEC-M, and 4OI@iEC-M. Briefly, membrane proteins were extracted by utilizing Membrane protein extraction kit (Beyotime biotechnology, Shanghai, China). After membrane proteins separated into different bands by electrophoretic separation, protein bands were stained by Coomassie blue method.

### ELISA assay

RAW 264.7 cells were grown in 24 well plates for 24 h. The supernatants were collected and tested for IL-6 and IL-10 expression levels using an ELISA kit following the manufacturer’s instructions.

### Tube formation assay

The HUVECs were grown in Endothelial Cell Medium (ECM). The high glucose and palmitic acid treatment was given with 30 mM glucose and 100 µM palmitic acid. HUVECs were seeded on Matrigel 356,234 pre-coated 96-well plates after being exposed to different treatments. After incubation on Matrigel for 6 h, tube formation of these cells was observed under a light microscope.

### Transwell assay

Treated HUVECs were seeded on the upper chamber of Transwell chamber plates, and the bottom well was filled with medium containing 20% FBS. After 24 h in the incubator at 37 °C, migrated HUVECs were fixed and stained, the average number of which was detected under a light microscope.

### Diabetic wound model

All animal procedures were performed according to the guidelines of the Institutional Animal Care and Use Committee of Huazhong University of Science and Technology (IACUC Number: 3116). C57/BL6 mice (male, 7 to 8-week-old) were kept in a specific pathogen-free (SPF) environment. Diabetic mouse models were induced using streptozotocin (STZ, Sigma). After the mice were anesthetized, a 1 cm-diameter circular full-thickness skin wound was made on the back. Then the mice were randomly assigned to five treatment groups: Control, GelMA, 4OI/GelMA, iEC-M/GelMA, and 4OI@iEC-M/GelMA. 100 µl hydrogel systems were spread out over the wound surface in each treatment group and crosslinked by utilizing UV light.

### Histological analysis

The skin tissues and organs were fixed immediately after removal, and were then progressively dehydrated and wrapped in paraffin. Sliced samples were used for further experiments (H&E, Masson, immunohistochemical, and immunofluorescent staining).

### Statistical analysis

The outcomes were expressed as mean ± SD and analyzed by GraphPad Prism software. One-way analysis of variance (ANOVA) and Student’s t-test were used to investigate the statistical significance (**p < 0.01, *p < 0.05).

### Electronic supplementary material

Below is the link to the electronic supplementary material.

Supplementary Material 1

## Data Availability

All data generated or analyzed during this study are available from the corresponding author.
