# Supplementary material for: Bioinspired nanovesicles released from injectable hydrogels facilitate diabetic wound healing by regulating macrophage polarization and endothelial cell dysfunction
Source: J Nanobiotechnology. 2023 Oct 3;21:358. doi: 10.1186/s12951-023-02119-3 (PMC10546738; doi:10.1186/s12951-023-02119-3)
Supplement: Supplementary file 1 — Supplementary Material 1 [file 12951_2023_2119_MOESM1_ESM.docx]

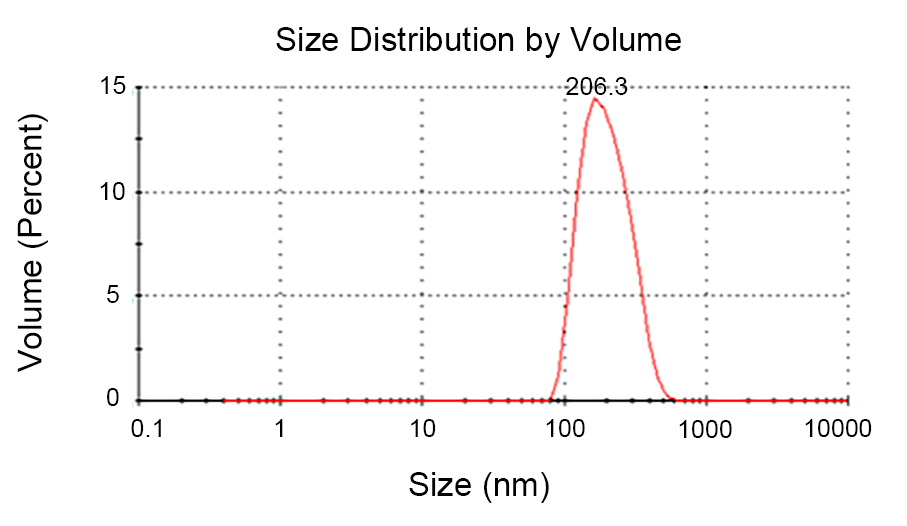


**Supplementary figure 1.** The dynamic light scattering (DLS) to analyze the size distribution of 4OI@iEC-M nanovesicles.


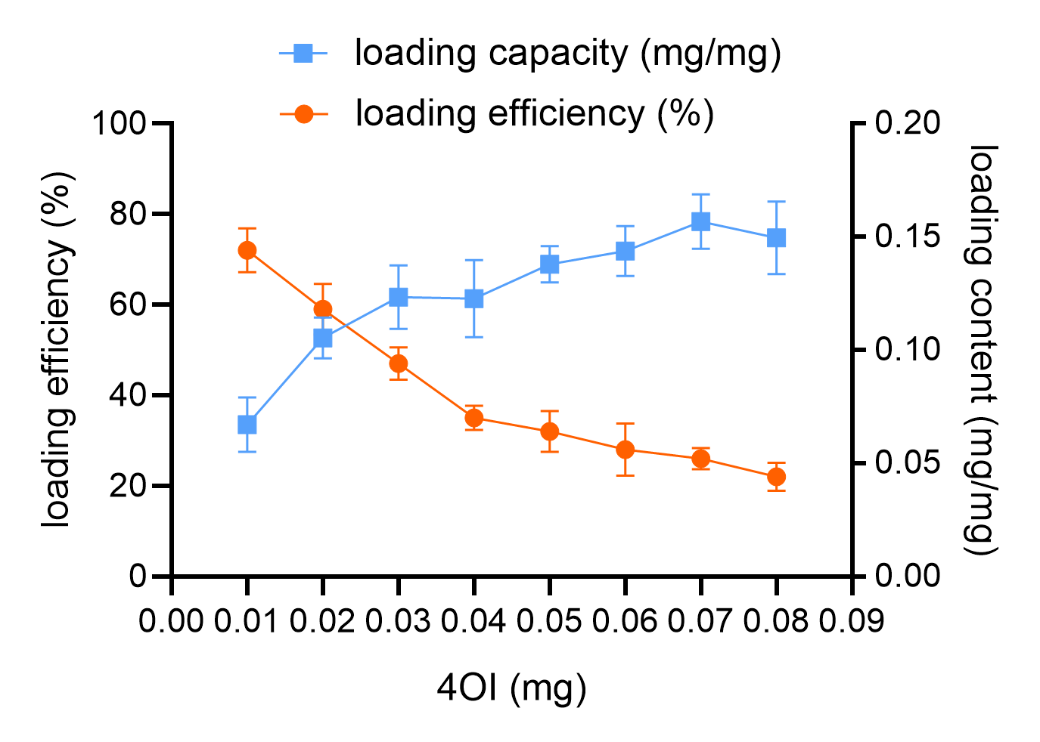


**Supplementary figure 2.** The drug loading efficiency and the drug loading capacity of 4OI@iEC-M were investigated.
